# Tissue adaptation to metabolic stress: insights from SUMOylation

**DOI:** 10.3389/fendo.2024.1434338

**Published:** 2024-11-11

**Authors:** Hao Xie, Xin Liu, Shuo Li, Ming Wang, Ying Li, Ting Chen, Linwei Li, Faxi Wang, Xuan Xiao

**Affiliations:** ^1^ Department of Clinical Laboratory, Institute of Translational Medicine, Renmin Hospital of Wuhan University, Wuhan, Hubei, China; ^2^ Department of Interventional Radiology, Renmin Hospital of Wuhan University, Wuhan, Hubei, China; ^3^ Department of Ophthalmology, Renmin Hospital of Wuhan University, Wuhan, Hubei, China

**Keywords:** post-translational modification, SUMOylation, metabolic homeostasis, obesity, insulin resistance

## Abstract

Post-translational modification (PTM) plays a crucial role in adaptation of mammals to environmental changes, enabling them to survive in stressful situations. One such PTM is SUMO modification, which is evolutionarily conserved. It involves the covalent and reversible attachment of a small ubiquitin-like modifier (SUMO) to lysine (Lys) residues in the target protein. SUMOylation regulates various functions, including cell proliferation, differentiation, apoptosis, senescence, and maintenance of specific cellular activities. It achieves this by influencing protein-protein interactions, subcellular localization, protein stability, and DNA binding activity. Mounting evidence suggests that SUMOylation is implicated in the pathogenesis of metabolic disorders such as obesity, insulin resistance, and fatty liver. This review aims to provide an overview of the role of SUMOylation in regulating tissue adaptation to metabolic stress. Recent advancements in spectroscopic techniques have shed light on potential targets of SUMOylation and the underlying regulatory mechanisms have been elucidated, laying the theoretical foundation for the development of targeted SUMOylation interventions for metabolic syndrome while minimizing side effects.

## 1 Introduction

**Figure 1:**
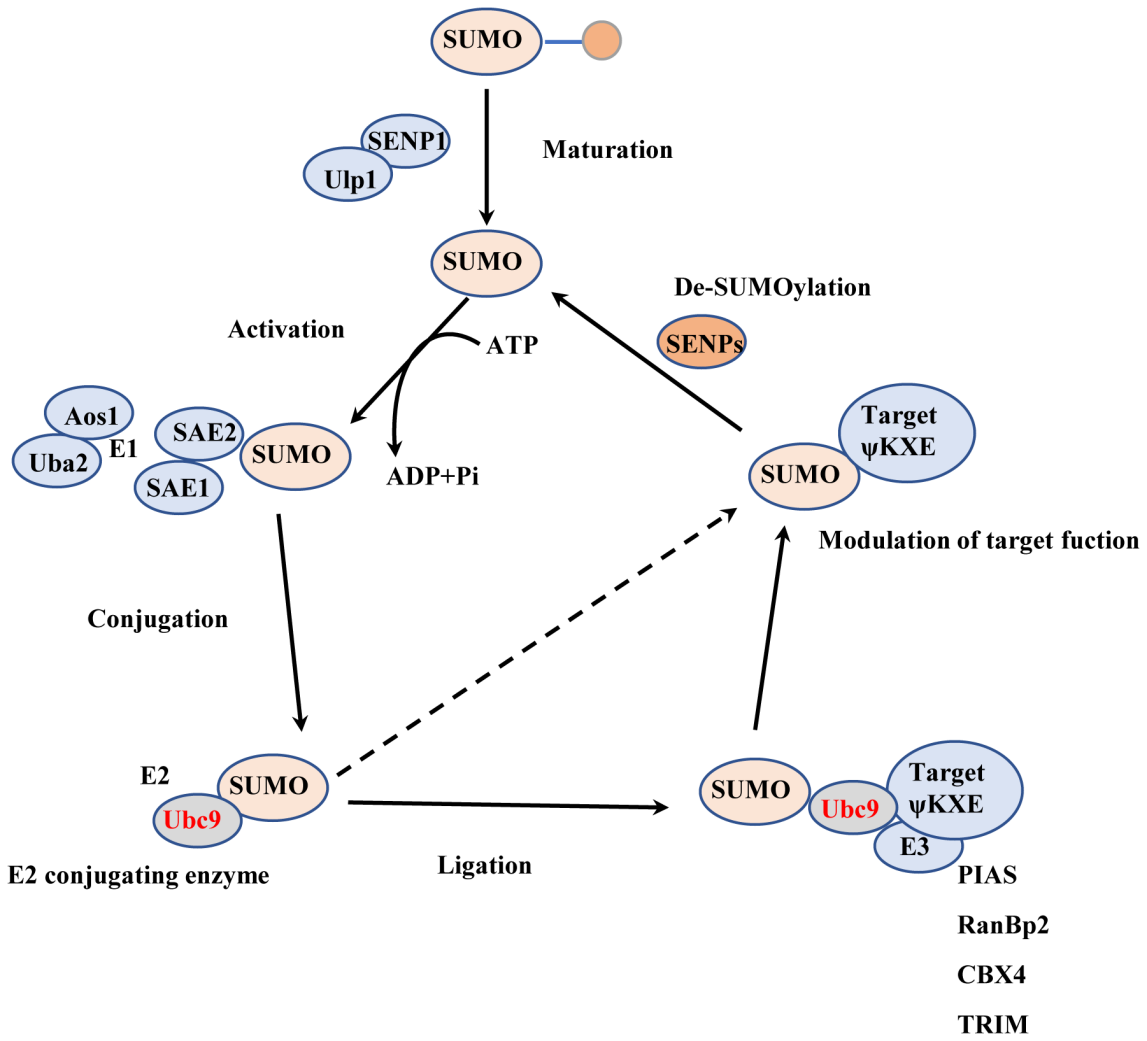
The process of SUMOylation. Maturation: The SUMO precursor molecule undergoes processing by ubiquitin-like specific protease 1 (Ulp1) or SUMO-specific protease (SENP), which removes the last four amino acids from the C-terminus, thereby exposing the terminal double glycine GG. Activation: The SUMO activating enzyme E1 (SAE1 or Aos1) and SUMO activating enzyme 2 (SAE2 or Uba2) usually form a heterodimer of SAE1-SAE2 or Aos1-Uba2. SAE2/Uba2 forms a thioester bond with the C-terminal carboxyl group of SUMO through its cysteine residue, thus activating the SUMO protein. Conjugation: The E2 conjugating enzyme Ubc9 forms an E2-SUMO thioester compound by establishing a thioester bond with the activated SUMO protein through its cysteine residue at position 93. Simultaneously. It attaches the SUMO molecule to the lysine residue of the target protein, completing the SUMOylation modification. Ligation: The SUMO E3 ligase promotes the detachment of the SUMO protein from Ubc9 by stabilizing the substrate and SUMO-E2 complex. It then attaches the SUMO protein to the substrate.

The process of SUMOylation involves the reversible covalent binding of small molecules from SUMO family to the lysine (Lys) residue in the target protein, under the action of E1 activating enzyme, E2, and E3 ligase (1). In mammalian cells, the SUMO family of small molecules comprises SUMO1, SUMO2/3, SUMO4, and SUMO5 (2–4). Among these, SUMO1 is the most extensively studied subtype. SUMO2 and SUMO3 share a high homology of 95% (5), but they only exhibit 45% homology with SUMO1 (6). Interestingly, both SUMO1 and SUMO2/3 possess very similar three-dimensional structures. SUMO4 is the least characterized SUMO subtype, and analysis of single nucleotide polymorphisms (SNPs) has revealed its association with type 1 diabetes (7, 8). In addition, SUMO4 expression is increased in the models of placental oxidative stress and hypoxia injury associated with pre-eclampsia (9). SUMO5 and SUMO4 have restricted expression to specific tissues, SUMO5 was found to regulate promyelocytic leukemia nuclear bodies (10, 11) (**Figure 1**).

The ATP-dependent heterodimer consisting of SUMO activating enzyme subunit 1 (SAE1) and SUMO activating enzyme subunit 2 (SAE2) plays a crucial role in activating the SUMO molecule and transferring the activated SUMO protein to a specific and unique E2 conjugating enzyme called Ubc9 (12, 13). Ubc9 typically works in conjunction with E3 ligases to facilitate the attachment of SUMO to the substrate (14). Several proteins with SUMO E3 activity, such as RanBP2, PIASs, Pc2, have been identified, and they enhance the binding of SUMO to the target protein (15, 16). The site structure for SUMOylation is ψ-K-X-E/D, where ψ is a hydrophobic amino acid, K denotes the lysine residue where modification occurs, X represents any amino acid, and D/E stands for Asp or Glu (17). SUMOylation is a reversible and dynamic process, and the modified protein can be deSUMOylated by SUMO-specific proteases known as SENPs. The SENP family comprises six members: SENP1-3 (18–20) and SENP5-7 (21–24). Different SENP members act on proteins bound to different types of SUMO molecules (25). SENP1 and SENP2 catalyze proteins bound to all types of SUMO molecules (26), while SENP3, SENP5, SENP6, and SENP7 preferentially remove SUMO2/3-bound proteins (27, 28). The process of SUMOylation shares similarities with ubiquitination, as both modifications occur on lysine residues of proteins (29). SUMOylation impacts ubiquitination-mediated protein degradation in a cooperative or competitive manner (30, 31). It also plays a role in regulating protein subcellular localization, protein-protein interactions, and protein-DNA binding. SUMOylation governs various functions of target proteins and interacts with other post-translational modifications, contributing to their irreplaceable roles in pathological and physiological conditions.

## 2 The role of non-covalent SUMO Interactions in cellular regulation

Non-covalent SUMO interactions involve proteins with one or multiple SUMO-interacting motifs (SIMs), distinct from covalent SUMOylation (32, 33). SIMs are characterized by short sequences rich in large hydrophobic residues like leucine, isoleucine, or valine, often flanked by acidic residues (34). These interactions can affect protein subcellular localization as well as nuclear events like transcription and chromosomal maintenance (35, 36), and are crucial in DNA repair processes such as XRCC4-mediated double-strand break repair (37).

## 3 The role of SUMOylation in physiology

SUMOylation serves as a pivotal mechanism in spermatogenesis, orchestrating critical processes such as meiotic sex chromosome inactivation, centromeric heterochromatin organization, XY body formation, and regulation of meiotic recombination in coordination with the ubiquitin-proteasome system (38, 39). Moreover, it targets an array of proteins involved in fundamental tasks like chromosome pairing and recombination, although the full functional implications of this modification await comprehensive elucidation. In mouse oocyte maturation, SUMOylation distinctly governs spindle organization, chromosome congression, and segregation, with SUMO1 and SUMO2/3 assuming discrete roles (40). The activity of deSUMOylases like SENP2, SENP3, and SENP7 is imperative for proper oogenesis, while in C. elegans, SUMO modification modulates chromosome congression (41–43). Overall, SUMOylation emerges as indispensable for the seamless progression of oocyte development and for effective communication with ovarian somatic cells.

In embryogenesis, SUMOylation assumes crucial roles. Early studies showed that the absence of SUMO1 results in embryonic developmental defects and embryonic death between E13.5-E18.5 (44). The lack of SUMO1 is also associated with high prenatal and postnatal lethality due to heart defects (45). However, other studies have reported that the SUMO1-deficient mouse model does not exhibit abnormalities under normal feeding conditions (46–48). Interestingly, mice lacking SUMO3 can survive, possibly due to the compensatory effect of SUMO2 (49). In contrast, the absence of SUMO2 cannot be compensated by SUMO3 or SUMO1, indicating that certain key proteins modified by SUMO2 are irreplaceable for maintaining cellular function (49). SUMO2 and SUMO3 are dynamically regulated in response to various cellular stresses like DNA damage (50, 51) or during cell cycle progression (52). Notably, the larger free pool of SUMO2 enables its conjugation under stress conditions, highlighting its significance in cellular stress response mechanisms (53).

Consistent with the pivotal role of SUMOylation in embryonic development, the deletion of the conjugating enzyme Ubc9 caused embryonic lethality in mice. additionally, mice lacking E3 ligases such as PIAS1 (54) and CBX4 (55) exhibit varying levels of perinatal death. Among them, the surviving *Pias1-/-* that survive are smaller than wild-type mice but do not display significant histological defects (54). They do, however, exhibit enhanced immune responses to viruses or microbes (54). Other factors with SUMO E3 ligase activity, such as RanBP2 or KAP1, are also essential for embryonic development (56, 57). Conversely, the knockout of the SUMO protease SENP1 or SENP2 also results in embryonic lethality during mid-embryo development. Overall, maintaining the balance of SUMOylation is indispensable for embryonic development and maintenance of organ function.

## 4 The role of SUMOylation in metabolic homeostasis

### 4.1 Liver

SUMOylation plays a significant regulatory role in hepatic cells and liver diseases, influencing various cellular processes such as the cell cycle, apoptosis, DNA repair, and signal transduction, and is crucial for maintaining hepatocytes function and addressing liver diseases. The development of hepatocytes requires the synthesis of new proteins in response to environmental stress, enabling the cells to acquire functions. Each stage of hepatic maturation is distinguished by a distinct expression pattern of liver- and stage-specific genes (58). Simultaneously, existing proteins can undergo post-translational modifications (PTMs), leading to structural changes and eliciting signal responses (59). During the differentiation human embryonic stem cell (hESC) into hepatocytes, the levels of SUMO1- and SUMO2-modified proteins was observed to decrease, concomitant with an increased expression of the SUMO-deconjugating enzyme SENP7 (60). Hepatocyte nuclear factor 4 alpha (HNF4α) is a key regulator of liver-specific genes involved in metabolism and coagulation (61–63). It controls hepatocyte differentiation and acts as a tumor suppressor, regulating cell growth (64). Loss of HNF4α leads to dedifferentiation and impaired liver regeneration after partial hepatectomy (65). DeSUMOylation of HNF4α is essential for hepatocyte maturation by preventing its ubiquitin-mediated degradation, thereby coordinating nutrient metabolism and managing intracellular stress, including xenobiotics and metabolic wastes (60, 66).

**Figure 2:**
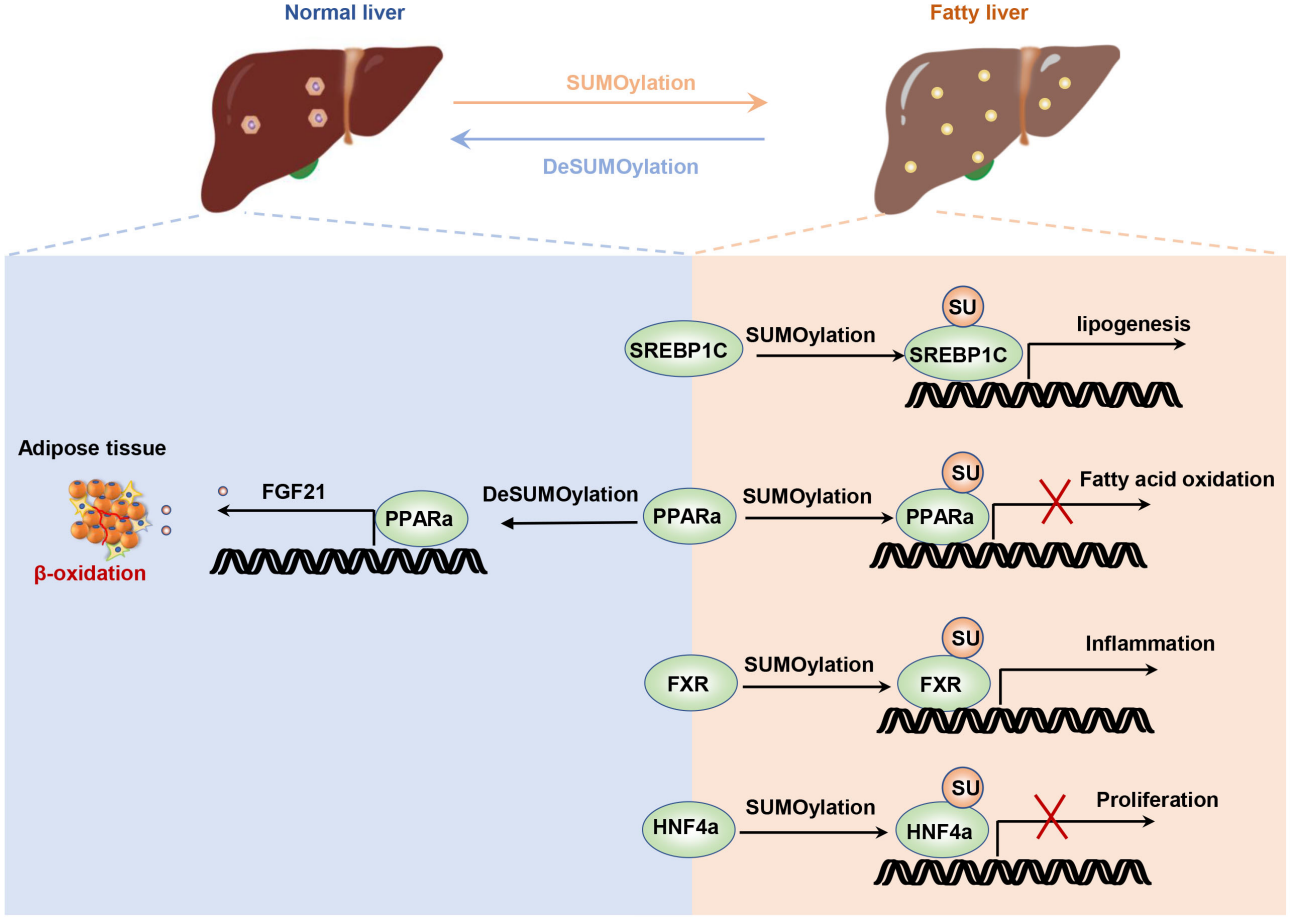
SUMOylation regulates liver energy metabolism. The SUMOylation of HNF4α inhibits hepatocyte proliferation and post-injury repair. The SUMOylation of SREBP1C suppresses its ubiquitination degradation pathway, thereby increasing lipid synthesis. The SUMOylation of FXR enhances its transcriptional activity on inflammatory factors. Additionally, disruption of PPARa not only enhances lipid oxidation in hepatocytes but also increases hepatic FGF21 secretion, which promotes lipid oxidation in adipocytes.

The imbalance between energy intake and expenditure is a common characteristic of metabolic diseases, including obesity, insulin resistance, and fatty liver (67). Non-alcoholic fatty liver disease (NAFLD), a prevalent liver metabolic disease, is characterized by lipid accumulation in the liver, leading to steatosis, inflammation, cirrhosis, and liver cancer (68). The liver, as the central regulatory system of metabolic homeostasis, is finely regulated. Sterol regulatory element binding protein 1c (SREBP1c) is a key transcriptional factor for lipogenesis, and its transcription and translation levels are strictly controlled (69, 70). SUMOylation of SREBP1c reduced its transcriptional activity and decreased the expression of lipogenic genes in the liver (71). PIASy promotes the SUMOylation of SREBP1c, resulting in the reduction of hepatic lipogenesis and alleviations of hepatic steatosis (30). Similarly, liver-specific knockout of *Senp2* in mice confers to resistance to hepatic steatosis and obesity induced by a high-fat diet. SUMOylation of peroxisome proliferator-activated receptor alpha (PPARα) decreased its ubiquitination-relative degradation, thereby enhancing fatty acid oxidation (72). PPARα and farnesoid X receptor (FXR) have opposite regulatory effects on liver lipid metabolism (73, 74). PPARα is activated during fasting and induces fatty acid oxidation, while FXR controls bile acid homeostasis and is activated during feeding (75). SUMO2 modification of FXR at K277 alleviated liver inflammation and lipid accumulation by reducing the expression of inflammatory gene expression regulated by NF-κB (76). It appears that promoting protein SUMOylation in hepatocyte enhances liver energy metabolism and alleviates metabolic stress (**Figure 2**).

Under metabolic stress, increased mitochondrial oxidative respiration and fatty acid oxidation exacerbate oxidative stress in hepatocytes, contributing to impaired liver function and disrupting the body’s energy metabolism (77). Hepatic ischemia/reperfusion-induced metabolic stress results from glucose or fatty acid energy substrates undergoing mitochondrial oxidation in the presence of sufficient oxygen, leading to cellular oxidative stress (78). SUMOylation is believed to play a role in maintaining the liver’s antioxidative capacity by regulating proteins associated with redox balance, such as Nrf2 (nuclear factor-2) (79). In particular, the deSUMOylation of sirtuin-3 (Sirt3) restores mitochondrial function, reduces oxidative stress, and prevents apoptosis induced by hepatic ischemia/reperfusion injury (80). Further research is needed to understand the precise role of SUMOylation in metabolic and pathological processes in the liver and to identify potential therapeutic targets.

### 4.2 Adipose tissue and adipocytes

Obesity is typically characterized by the expansion of adipose tissue and ectopic fat deposition, leading to various metabolic diseases such as insulin resistance, fatty liver, and cardiovascular diseases (81, 82). Adipose tissue is not only the largest energy reservoir in mammals but also an endocrine organ capable of synthesizing various bioactive secretory factors that regulate metabolic homeostasis. In mammals, adipose tissue is primarily composed of mature adipocytes, microvessels, and a complex stromal vascular fraction (SVF), which can be classified into two main types: white adipose tissue (WAT) and classic brown adipose tissue (BAT). White adipocytes, with a single large lipid droplet, are involved in lipid storage, mobilization, secretion of adipokines and immunoregulation (83–85). In contrast, distinguished by multilocular lipid droplets and abundant mitochondria, specialized in energy expenditure and adaptive thermogenesis (86, 87). Another type of adipocyte, known as beige or brite adipocytes, shares similarities with brown adipocytes and originates from smooth muscle-like progenitor cells or white adipocytes. Interestingly, alterations in the expression or activity of specific SUMO system components regulate the fate of adipocytes and the function of adipose tissue (88).

**Figure 3:**
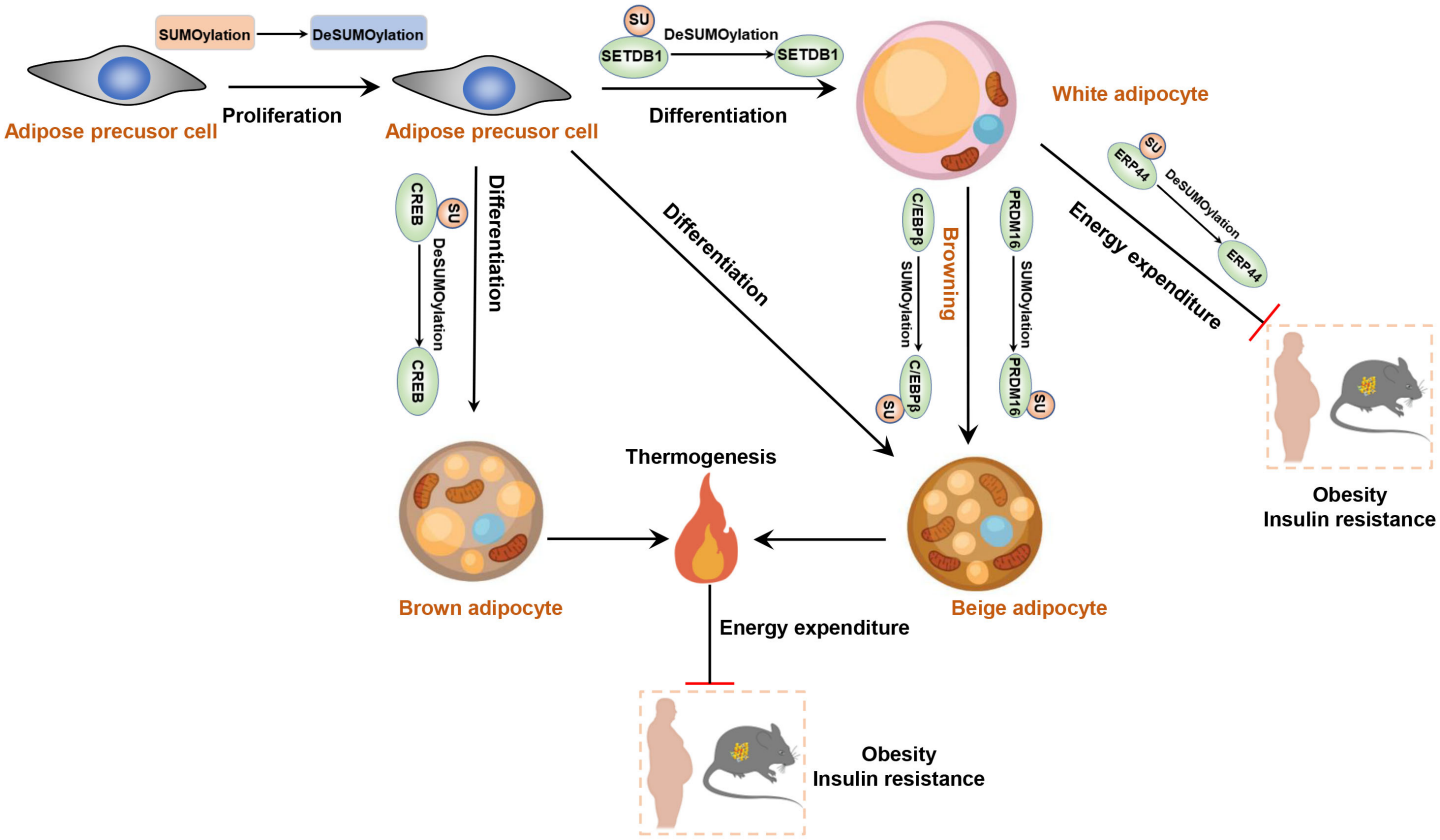
SUMOylation regulates adipocyte function and plays role in the development of obesity and insulin resistance. Reducing the level of SUMOylation increases the proliferative and differentiative capacities of adipose precursor cells. DeSUMOylation of SETB1 enhances the differentiation of adipose precursor cells into white adipocytes. Moreover, DeSUMOylation of CREB promotes the production of brown adipocytes. However, SUMOylation of C/EBPβ enhances the white-to-beige adipose transition.

For example, Knockdown of *Senp1* leads to reduced levels of key adipogenic regulators, such as C/EBP-α/β and PPARγ, impairing the migratory and proliferative capacities of human adipose-derived stem cells (hADSCs) and promotes apoptosis, ultimately inhibiting adipocyte differentiation (89, 90). Additionally, the SUMOylation of SETDB1 promotes its binding to the promoter region of PPARγ and C/EBPα, thereby reducing the lipid storage capacity of adipocytes (91). The small ubiquitin-like modifier-conjugating enzyme Ubc9 also plays critical role in adipocyte differentiation, as demonstrated by the fluctuation in its expression during the differentiation of adipose precursor cells into mature adipocytes (92, 93). Interestingly, adipocyte-specific depletion of *Ubc9* protects mice from high fat diet (HFD)-induced obesity and insulin resistance (94). In this context, SUMOylation of ERp44 aggravates ER stress in adipocytes, contributing to the development of obesity and insulin resistance, partly by increasing Ero1α retention in ER. Furthermore, the differentiation of brown adipocytes is also influenced by SUMOylation, as demonstrated by changes in SENP2 expression throughout the differentiation process (95). SENP2 catalyzes the de-SUMOylation of cAMP response element-binding protein (CREB), thereby promoting the differentiation of brown preadipocytes (96). Activating brown adipose tissue (BAT) or promotion of white-beige transition is a promising strategy for the treatment of obesity and type 2 diabetes mellitus (97, 98). Cold exposure or treatment of β-adrenergic receptors (β-ARs) agonist can induce the emergence of beige adipocytes from smooth muscle-like progenitor cells or white adipocytes (99–101). It is noteworthy that SUMOylated C/EBPβ promotes the formation of beige adipocytes in subcutaneous adipose tissue by downregulating the expression of the inhibitory gene HOXC10 (102). Additionally, the SUMOylation of PRDM16 is induced by exposure to cold environments and is also essential for white fat browning (103). In summary, SUMOylation is involved in multiple crucial biological processes in adipocytes and adipose tissue, and its dysregulation may be associated with the occurrence and development of obesity and related metabolic diseases. A thorough understanding of the molecular mechanisms of SUMOylation and its specific effects on obesity holds the potential to provide a theoretical basis for the development of novel therapeutic strategies in the future (**Figure 3**).

### 4.3 Islet

Pancreatic β cells, originating from the pancreatic primordium during embryonic development, play a crucial role in synthesizing and releasing insulin to regulate blood glucose levels (104). In diabetic individuals, pancreatic β cells may undergo reduction, atrophy, or functional abnormalities, rendering them incapable of effectively responding to glucose stimuli and causing elevated blood glucose levels (105). SUMOylation plays various crucial roles in pancreatic islet cells, involving physiological regulation, insulin secretion, and the survival and apoptosis of pancreatic islet cells. For instance, MafA is a β-cell-restricted basic leucine-zipper transcriptional activator, whose transactivator domain is SUMOylated, a process enhanced by PIASy (106). The interaction between MafA and PIASy requires the basic domain of MafA and the amino-terminal region of PIASy, which contains the SAP domain. Furthermore, the SUMO-interacting motif 1 (SIM1) located in the carboxyl-terminal region of PIASy is essential for repressing the synergistic transactivation of MafA along with other key transcription factors, Pdx1 and Beta2, which are critical for β-cell differentiation and function (107). Additionally, Kv2.1 channels contribute to maintaining the density of membrane-associated insulin granules and the number of fusion “hotspots”. SUMOylation of Kv2.1 occurs at the N-terminal (K145) and C-terminal (K470) sites, potentially regulating the relative proportion of fusion events within specific regions (108, 109).

Moreover, previous studies have indicated that SENP2 expression is upregulated in the islets of T2D animal models and patients, and chronic glucose stimulation also increases SENP2 expression in INS1 cells (110). SUMOylation of DRP1 due to SENP2 deficiency impairs its phosphorylation, leading to mitochondrial dysfunction and reduced insulin secretion in pancreatic β cells (111, 112),. Thus, investigating changes in mitochondrial function may be a key direction for studying SUMOylation in the physiological function of pancreatic β cells.

**Figure 4:**
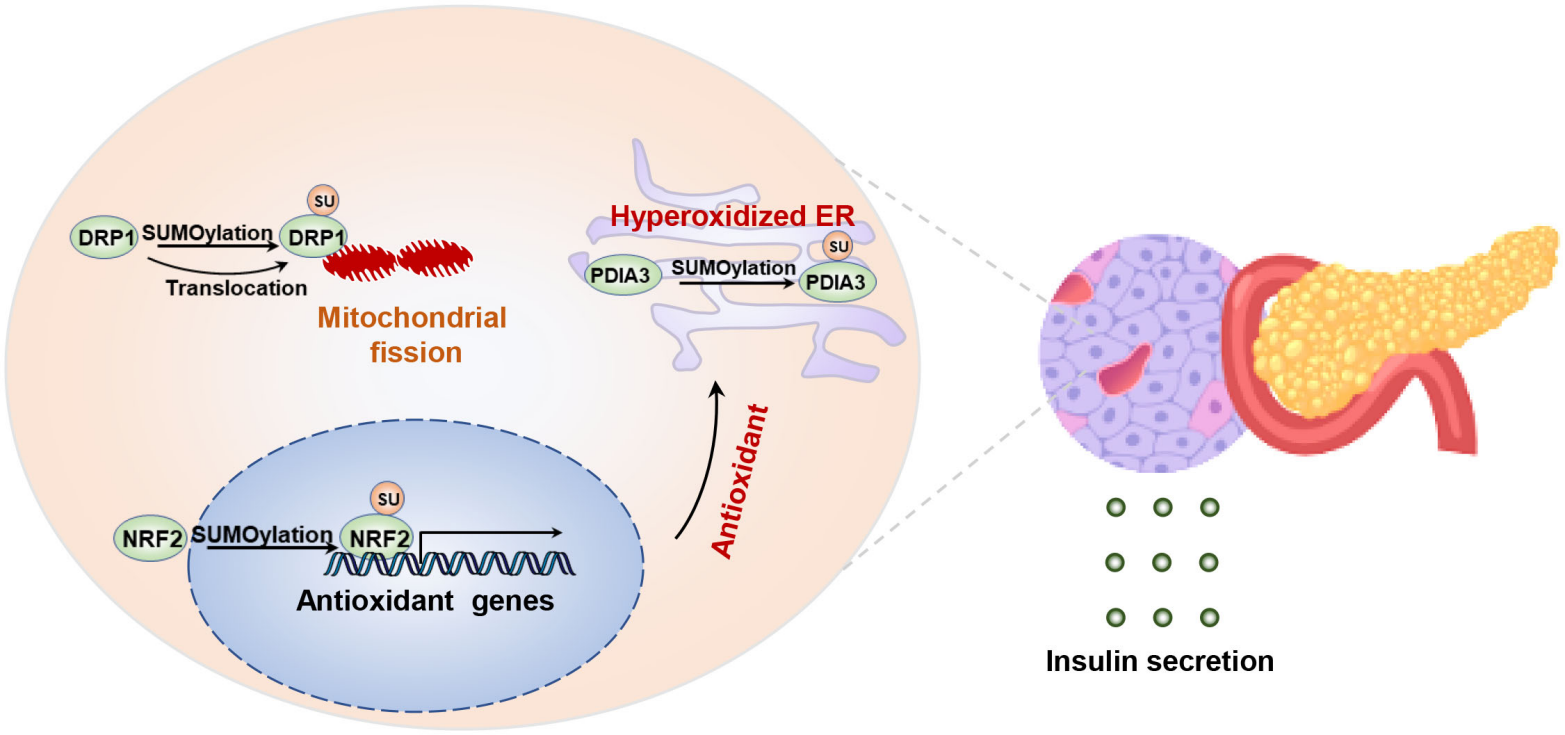
SUMOylation regulates islet β cell integrity and insulin secretion. Maintaining the balance between SUMOylation and deSUMOylation is crucial for preserving the stability of endoplasmic reticulum and mitochondrial function in β cells. SUMOylation of NRF2 increases the transcription of antioxidant genes, while SUMOylation of DPIA3 exacerbates oxidative folding and ER stress. Disruption of DRP1 SUMOylation leads to a reduction in mitochondrial fission by inhibiting the translocation of DRP1 to the mitochondria.

SUMOylation may participate in regulating the survival and apoptosis of pancreatic islet cells. Persistent high blood glucose level increases oxidative stress and endoplasmic reticulum stress in pancreatic beta cells, which can lead to cell apoptosis or transdifferentiation, consequently reducing insulin secretion (113, 114). Previous studies have shown that cytokine stimulation induces endoplasmic reticulum stress, accompanied by alterations in the SUMOylation profile in mouse/human pancreatic β cells (115). Specifically, SUMOylation of disulfide isomerase a3 (Pdia3) exacerbates proinsulin misfolding and endoplasmic reticulum stress (115). it seems that increased SUMOylation aggravates beta cell apoptosis while impairing insulin secretion (116). Conversely, the loss of Ubc9 increased oxidative stress in pancreatic beta cells, resulting in spontaneous diabetes in the mice (117). Because deSUMOylation of NRF2 suppresses its nuclear translocation and reduces the expression of its downstream antioxidant genes, resulting in accumulation of reactive oxygen species (ROS) (117). In short, both upregulation of downregulation of SUMOylation, through overexpression or ablation of *Ubc9*, induces pancreatic beta cells death and dysfunction (117). In essence, maintaining the dynamic balance of SUMOylation in β cells is crucial for transitioning from cell survival to secretory function (**Figure 4**).

### 4.4 Skeletal muscle

It is generally accepted that appropriate exercise can alleviate insulin resistance in patients with obesity or type 2 diabetes, primarily by enhancing skeletal muscle glucose uptake and fatty acid oxidation (118, 119). Skeletal muscle fibers possess a unique SUMO modification system involved in regulating the transition between different fiber types in terms of contractile and metabolic properties (120). The levels of SUMOylation and its substrates are strongly correlated with fiber type and exhibit significantly change before and after exercise (121). Recent studies have also reported the SUMOylation of α-actin in rat skeletal muscle (122). Intense muscle contractions promote the nuclear translocation of SUMO1 in human myofibres suggesting that SUMOylated proteins may be participate in the modulation of contractility (123).

**Figure 5:**
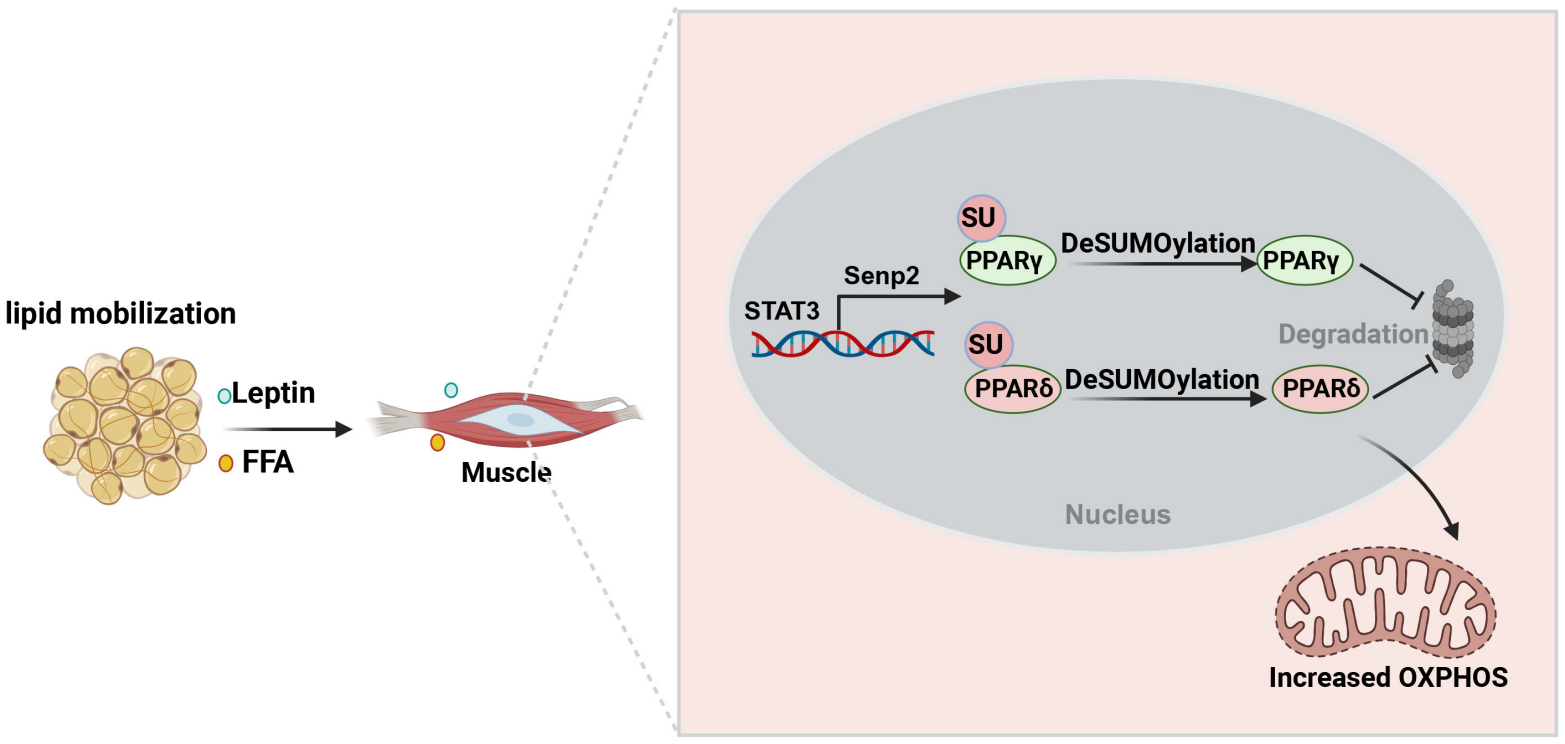
The role of SUMOylation in enhancing skeletal muscle plasticity and improving insulin resistance during exercise. Exercise promotes the mobilization of fat to produce free fatty acids that provide energy to skeletal muscles. Meanwhile, free fatty acids and leptin increase the expression of SENP2 in skeletal muscle. Reducing PPARδ and PPARγ SUMOylation enhances fatty acid oxidation, thereby increasing energy expenditure to promote weight loss and improve insulin sensitivity.

Although the effects of exercise on glucose uptake and oxidation in myotubes from individuals with obesity compared with lean individuals are inconsistent (124–126). Effects of exercise on obesity *in vivo* results from a combination of mechanical, metabolic and oxidative perturbations (127). During prolonged exercise, fat mobilization is the main source of the free fatty acids (FFAs) for muscle contraction (128, 129). FFA increase the expression of SENP2 in myotubes, leading to the up-regulation of fatty acid oxidation-related enzymes via decreasing SUMOylation of PPARδ and PPARγ (130). Similarly, treatment C2C12 muscle cells with palmitate induces the expression of SENP2 and enhances oxidation of fatty acids through toll-like receptor (TLF) 4/MyD88/NF-κB signaling pathway (130–132). Overexpression of muscle-specific *Senp2* alleviates high-fat diet-induced obesity and insulin resistance (132). In addition, leptin activates the binding of STAT3 to the promoter region of SENP2 and promotes its expression, indicating the synergistic effect of adipose tissue and skeletal muscle in regulating the body’s energy homeostasis (130). In conclusion, in view of the fact that increasing the SUMOylation in skeletal muscle can enhance energy expenditure, inhibiting SENP2 expression could serve as a novel therapeutic approach to alleviate obesity and hyperlipidemia (**Figure 5**). Actually, SUMOylation may play a role in regulating proteins related to muscle contraction and movement, such as myosin and troponin (133). A thorough understanding of the molecular mechanisms of SUMOylation in these processes is of significant importance for comprehending muscle biology and the pathogenesis of metabolic diseases.

### 4.5 Nervous system and gastrointestinal system

SUMOylation plays a crucial role in regulating appetite in the nervous system and nutrient absorption in the gastrointestinal system. In the nervous system, SUMOylation may regulate appetite through modulating the activity of neurons and influencing neurotransmitter systems such as norepinephrine and dopamine (134–136). Additionally, SUMOylation can impact interactions between neurons, potentially by altering the SUMOylation status of proteins, thereby regulating synaptic transmission and neuronal network activity (137, 138). In the gastrointestinal system, SUMOylation primarily acts by modulating proteins related to nutrient absorption and metabolism (139, 140). This involves affecting the stability of proteins and regulating cell signaling pathways, such as insulin signaling (141). Furthermore, SUMOylation may regulate the secretion of hormones that play a role in the gastrointestinal system, such as glucagon-like peptide-1 (GLP-1) and gastric hormones (142). In summary, SUMOylation plays a critical role in regulating appetite and nutrient absorption in both the nervous system and gastrointestinal system. These processes involve complex molecular mechanisms that require further in-depth research to understand their mechanisms of action and biological effects.

## 5 Discussion and prospective

**Figure 6:**
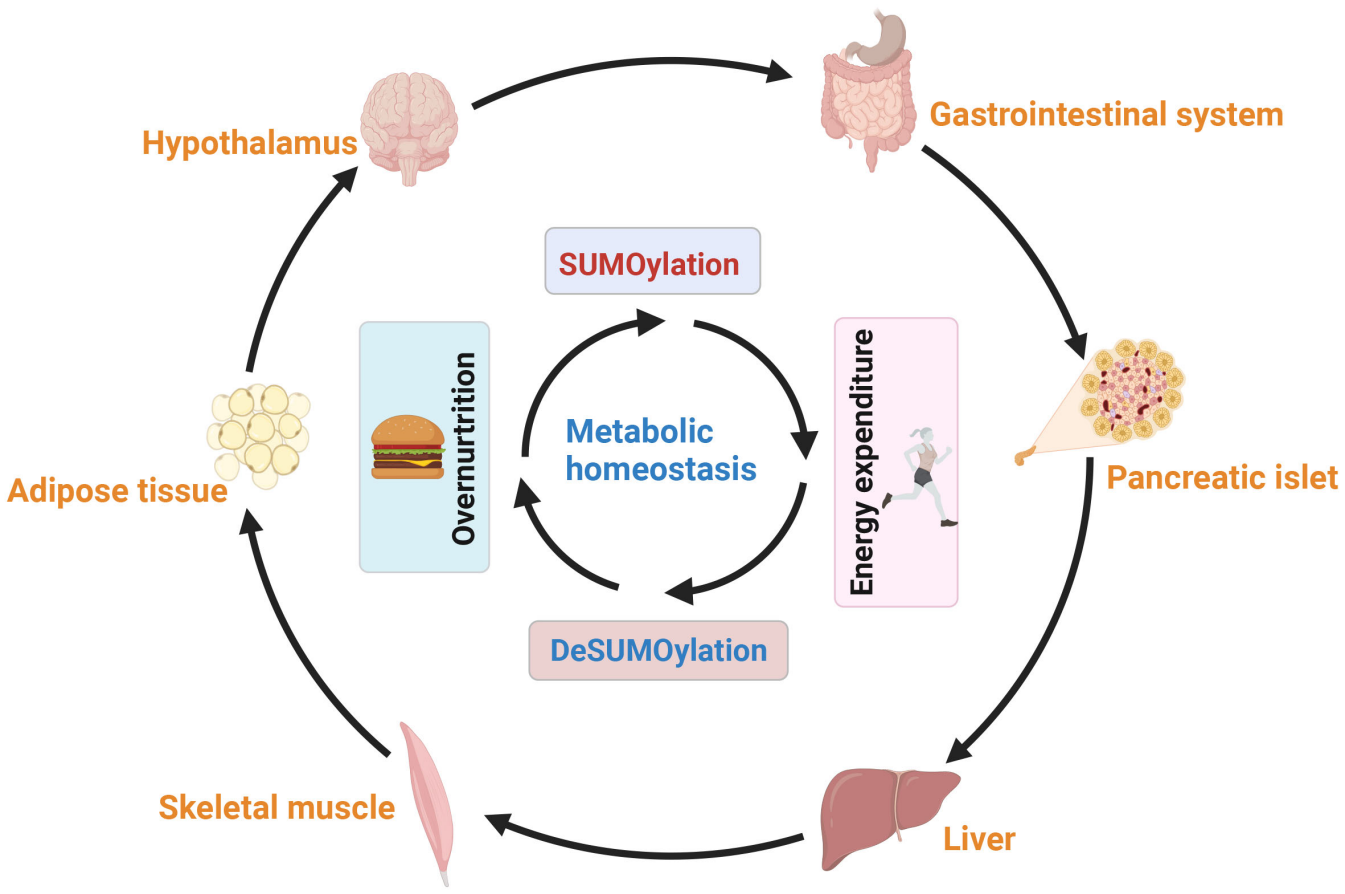
The balance between SUMOylation and deSUMOylation maintains energy metabolism homeostasis. SUMOylation impacts neural proteins, including neurotransmitter receptors and appetite-controlling neurons, modulating their activity and influencing appetite regulation, hunger, and eating behavior. Additionally, SUMOylation affects hormone secretion and gastrointestinal motility, regulating food absorption and satiety. SUMOylation also plays a key role in insulin secretion and metabolic tissues (liver, adipose, and muscle), facilitating communication through secreted factors to maintain overall energy balance. In summary, SUMOylation regulates neural, appetite, gastrointestinal, and metabolic interactions, ensuring energy balance across various tissues and metabolic states.

Broadly speaking, the interplay between with liver, adipose tissues, muscle, islet, nervous and gastrointestinal system plays a central role in the regulation of systemic glucose and lipid fluxes during feeding and fasting. Research has highlighted the tremendous potential of SUMOylation in modulating metabolism and endocrine function of these tissues to maintaining systemic energy balance and metabolic homeostasis (**Figure 6**). Disruption the balance of SUMOylation between deSUMOylation can lead to the occurrence of metabolic syndrome, including NAFLD, cardiovascular disease, obesity associated diseases, and cancer. However, the diverse components of the SUMO system sometimes yield contradictory results due to their distinct roles in specific pathophysiology. Therefore, it is important not to generalize the regulatory role of SUMO modification in different cells under the same pathological conditions

To further investigate the role of SUMOylation in pathophysiology, researchers have established mouse models with inducible knockout or overexpression of *Ubc9*, a key enzyme involved in SUMOylation. The effects of SUMOylation can vary significantly across different metabolic environments and cell types, primarily due to the diversity of SUMO molecules and their substrates. The effects of SUMOylation can vary significantly across different metabolic environments and cell types due to the diversity of SUMO molecules and their substrates. However, as the sole E2 conjugating enzyme for SUMOylation, Ubc9 serves as a pivotal point for understanding the overall regulatory role of this modification. Importantly, *Ubc9* can be selectively depleted in different tissues through the administration of tamoxifen to adult mice, allowing for the exploration of SUMOylation’s role in regulating cellular functions (94, 143).Investigating *Ubc9* knockout models offers a comprehensive perspective on the functional significance of SUMOylation, as the deletion of *Ubc9* eliminates all SUMO modifications. For instance, research using Ubc9 KO mice has revealed that the loss of Ubc9 leads to a marked increase in oxidative stress in pancreatic beta cells due to impaired NRF2 activity, which is crucial for ROS detoxification (117). Additionally, *Ubc9* deletion in macrophages has been shown to disrupt the M2 macrophage activation program (144), exacerbating type 1 diabetes progression through enhanced T cell activation (145). This approach allows us to discern the collective impact of SUMOylation on various cellular processes without the compensatory effects seen with individual SUMO proteins. By the way, Ubc9 is found in both the nucleus and cytoplasm, including the endoplasmic reticulum and mitochondria, but its potential role in regulating metabolic function independently of SUMOylation remains unclear. Further research is needed to fully explore the additional features of this protein.

The study of SUMOylation has lagged behind the discovery of other post-translational modifications, such as phosphorylation and ubiquitination. However, approaches for the enrichment of endogenous wild-type SUMO-modified peptides and the identification of SUMOylation sites remain limited. Continuous advancements in analytical methods are essential for enriching our understanding of the spatiotemporal characteristics of SUMOylation and pinpointing specific SUMOylated sites. For instance, Francis Impens et al. generated SUMO1 T95R and SUMO2 T91R variants, which enabled the identification of SUMO-modified peptides via classical LC-MS/MS following trypsin digestion (146). Cai et al. utilized a high-affinity SUMO1 antibody to facilitate the enrichment of SUMO1-modified peptides, leading to the identification of 53 high-confidence SUMO1-modified sites in mouse testis (147). Additionally, peptide-level immunoprecipitation has proven effective, allowing for the identification of 14,869 endogenous SUMO2/3 sites in human cells under stress conditions, while quantitatively mapping 1963 SUMO sites across various mouse tissues (17). Moreover, WaLP digestion has produced peptides with KGG remnants at the SUMO modification sites, facilitating the identification of 1209 unique SUMO modification sites under native conditions (148). The K0 strategy, which involved His10-tagged SUMO with all lysines substituted by arginines, has further enhanced this field by preventing digestion by the endoproteinase Lys-C and allowing for stringent purification and improved identification of SUMOylation sites (149). In their study, Michael et al. employed an augmented K0-SUMO proteomics technique to identify an impressive 40,765 SUMO acceptor sites and assess their impact on 6,747 human proteins. In their study, Michael et al. employed an augmented K0-SUMO proteomics technique to identify 40,765 SUMO acceptor sites and assess their impact on 6,747 human proteins (53). They uncovered 807 SUMOylated peptides co-modified by phosphorylation and detected co-modifications with ubiquitylation, acetylation, and methylation. Particularly noteworthy was the finding that 9% of the SUMOylome was in proximity to phosphorylation, with certain SUMOylation sites reliant on prior phosphorylation events (53). Collectively, these methodologies not only provide deeper insights into SUMOylation but also pave the way for a more comprehensive understanding of the dynamic interplay between various post-translational modifications, ultimately enriching our knowledge of protein function and regulation. For example, phosphorylation of SREBP1c by PKA enhances its SUMOylation, which in turn promotes ubiquitin-mediated degradation of SREBP1c (30). Post-translational modifications, including phosphorylation and SUMOylation, intricately regulate Drp1’s activity and mitochondrial localization, governing mitochondrial fission dynamics (150). The S616A mutant, which represents the non-phosphorylated state of Drp1, showed increased SUMOylation compared to the wild type, while the S616D mutant, representing the constitutively phosphorylated state, exhibited decreased SUMOylation (151). Additionally, the non-SUMOylated Drp1-4KR mutant increased Ser616 phosphorylation, and inhibiting SUMOylation also enhanced this phosphorylation, suggesting a complex regulatory mechanism between Drp1 phosphorylation and SUMOylation SUMO1 conjugation stabilizes Drp1’s association with mitochondria, promoting mitochondrial fragmentation and apoptosis (152). Interestingly, metabolites not only function as energy carriers but also serve as signaling molecules crucial in the onset and progression of metabolic disorders. The post-translational modification of proteins by metabolites, encompassing acetylation, palmitoylation, succinylation, and lactylation, involves intricate interactions within multiple metabolic pathways. Such investigations are poised to further elucidate the interconversion between various post-translational modifications, expanding the chemical space and functional repertoire of proteins through PTMs on diverse amino acid residues.

Current therapeutic strategies targeting the SUMOylation pathway, including E1 inhibitor TAK-981 (52), E2 inhibitor 2-D08 (153), and SENP1 inhibitor (154), modulate overall SUMOylation levels but may cause side effects. Regarding the safety and potential side effects of targeted SUMOylation interventions, most research has primarily focused on cancer and other diseases, with limited application in metabolic syndrome. In our unpublished study, we developed competitive peptides targeting SUMOylation-modified sites, using cell-penetrating peptides to inhibit abnormal SUMOylation without affecting normal protein function or overall SUMOylation, thus minimizing side effects. To further mitigate these side effects, future studies must deepen our understanding of the specific *in vivo* functions of SUMOylation, particularly in metabolic diseases. Unlike GLP-1 receptor agonists, which have well-established clinical safety data for treating metabolic syndrome, SUMOylation interventions lack extensive validation. Developing selective SUMOylation inhibitors with safety profiles similar to GLP-1 agonists could enhance their clinical value. Current trials of TAK-981 and other SUMO inhibitors in cancer provide a reference for future use in metabolic disorders. Advances in catalytic site and protein structure design techniques also offer promise for developing selective inhibitors, highlighting the need for further clarification of SUMOylation’s regulatory mechanisms.
